# An Artificial Cofactor Catalyzing the Baylis‐Hillman Reaction with Designed Streptavidin as Protein Host**

**DOI:** 10.1002/cbic.202000880

**Published:** 2021-02-16

**Authors:** Horst Lechner, Vincent R. Emann, M. Breuning, Birte Höcker

**Affiliations:** ^1^ Department of Biochemistry University Bayreuth Universitätsstrasse 30 95447 Bayreuth Germany; ^2^ Organic Chemistry University Bayreuth Universitätsstrasse 30 95447 Bayreuth Germany

**Keywords:** artificial cofactors, Baylis-Hillman reaction, biocatalysis, protein design, streptavidin

## Abstract

An artificial cofactor based on an organocatalyst embedded in a protein has been used to conduct the Baylis‐Hillman reaction in a buffered system. As protein host, we chose streptavidin, as it can be easily crystallized and thereby supports the design process. The protein host around the cofactor was rationally designed on the basis of high‐resolution crystal structures obtained after each variation of the amino acid sequence. Additionally, DFT‐calculated intermediates and transition states were used to rationalize the observed activity. Finally, repeated cycles of structure determination and redesign led to a system with an up to one order of magnitude increase in activity over the bare cofactor and to the most active proteinogenic catalyst for the Baylis‐Hillman reaction known today.

## Graphical abstract

**A story in pictures**: An artificial cofactor based on an organocatalyst was embedded in streptavidin to catalyze the Baylis‐Hillman reaction. The following protein design process was described by solved crystal structures for every design step, and the outcome of the chemical reaction was represented by using DFT calculations and MD simulations.

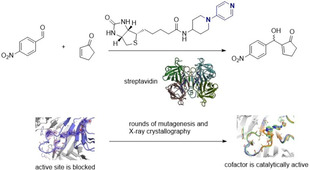

The design of proteins that display new catalytic activities is still a major challenge. Although several successful examples were reported,[^1^, ^2^, ^3^] these *de novo* cases are limited to a small set of reactions, such as the Kemp elimination,[^4^, ^5^] the retro‐aldol reaction,^[6]^ and a bimolecular Diels‐Alder reaction.^[7]^ All the initial designs provided a sufficient starting point (*k*_cat_ 0.17) but had to be strongly improved through directed evolution to show reasonable rate enhancement.[^8^, ^9^, ^10^]

Half of all natural enzyme catalyzed reactions require cofactor(s) as part of their catalytic machinery.^[11]^ Therefore, it is not surprising that far more new protein‐based catalysts were reported using artificial (metal‐based) cofactors embedded in host‐proteins.[^12^, ^13^, ^14^, ^15^, ^16^] These cofactors bear the advantage of intrinsic activity, which is usually low without a surrounding protein. The protein can provide a different environment for the catalysis – in most cases increasing the activity and maybe even facilitating (stereo)selective reactions.

There are several examples using streptavidin as the host for these cofactors, since its natural ligand biotin binds strongly (*K*_d_∼10^−15^ M) and is easy to modify with catalysts at the carboxylic acid group. The location of the catalyst is in a shallow cavity at the surface of the protein. Streptavidin is a known thermo‐ and solvent stable protein, which is another advantage in the development of new catalysts. Contrary to metal‐based cofactors, organocatalysts are rarely used in this context. There are only few examples: proline^[17]^ and an imidazolium salt^[18]^ were applied in wild‐type (wt)‐streptavidin as host for Michael additions and Aldol reactions, respectively. Additionally there are examples for organocatalysts embedded into proteins via noncanonical amino acids.[^19^, ^20^]

No enzyme is known to naturally catalyze the Baylis‐Hillman reaction,[^21^, ^22^] a very versatile and atom‐economic C−C bond forming reaction for the production of various functionalized compounds^[23]^ and intermediates of active pharmaceutical ingredients (APIs).^[24]^ There were attempts to design a *de novo* enzyme but with only minor success, leading to 24 % yield using 38 Mol% catalyst (BH32 N14I) after 28 hours.^[25]^ Nevertheless, it was recognized that this reaction can be catalyzed using nucleophilic amine catalysts in aqueous systems, although with low rates and high catalyst concentrations. (Imid)azoles,[^26^, ^27^] DABCO,[^28^, ^29^] 3‐hydroxyquinuclidine^[29]^ and 4‐dimethylaminopyridine (DMAP)^[29]^ were reported as catalysts for this purpose. We describe here the use of an artificial cofactor employing the well‐known biotin‐streptavidin system to catalyze a Baylis‐Hillman reaction.

We chose a derivative of DMAP as the artificial cofactor due to its low steric demand, which will facilitate its inclusion into the protein scaffold. Starting from 4‐(4‐aminopiperidino)pyridine we successfully synthesized the artificial cofactor **4** (Figure 1B).

**Figure 1:**
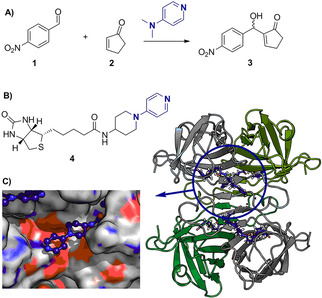
Concept of the artificial cofactor‐protein host system. A) model reaction: DMAP‐catalyzed Baylis‐Hillman reaction between enone **1** and aldehyde **2** providing racemic alcohol **3**. B) Artificial cofactor **4** consisting of a biotinylated DMAP derivative. C) Crystal structure of the tetrameric wt streptavidin harboring **4** and a closeup of the binding site of **4**.

A streptavidin variant (PDB ID: 5F2B),^[30]^ named here wt streptavidin, was used as the protein host due to its aforementioned advantages. The protein was expressed, purified and, after adding **4**, its activity tested in the Baylis‐Hillman model reaction with *p*‐nitrobenzaldehyde **1** and cyclopentenone **2** as the substrates (Figure 1A). No activity above the background activity as catalyzed by streptavidin itself (Table 1, entries 4 and 5) could be detected. To reveal the reason why there was no catalytic activity, the protein was crystallized and the X‐ray structure was solved (Figure 1C, PDB ID: 6T1E). It clearly visualized that **4** is placed nicely at the entrance of the biotin‐binding pocket. The density of the ligand is very well resolved (see Composite Omit maps, Figure S1 in the Supporting Information). As depicted in Figures 1C and 2A, the active site of the catalyst is sterically hindered by the side chains of Q114 and R121 and its position will be mainly affected by S112 at the bottom of the pyridine part. Especially position 121 is known to influence the outcome of metal‐based catalysts to a great extent.^[31]^ Thus, to reduce the steric hindrance at the catalytic site, bulky Q114 was changed to A or T, and R121 and S112 were substituted by A as well to create space at the active site, and to position the catalyst as deeply as possible in this cleft. Additionally, the variant S112A, R121A was tested, but none of these variants showed activity higher than the background. Exemplarily the activity of streptavidin S112A Q114A R121A is shown in Table 1, entry 6. The derived crystal structures of these variants revealed that **4** was now shifted to a position close to the amide carbonyl oxygen of A121, leading to an inaccessible nucleophilic nitrogen atom (Figure 2B yellow). An attempt of introducing Y as a bulkier amino acid at position L124 behind the catalyst to push the DMAP nitrogen atom forward was not successful either (Figure 2B pink). In a next step we varied S112 beneath the catalyst. By increasing its size from A to its original S and further increasing this residue to M, F and I, we reasoned it might force the catalyst into a more productive position. The variant harboring S112, however, led to no activity and a similar structure was obtained (PDB ID: 6T30) as in round 1 and 2 of our design approaches. But in contrast, all variants harboring sterically more demanding residues at position 112 (I, F, M) displayed activity (Table 1, entries 7–11 and Table S7, entries 4–15). Our reasoning was supported by the X‐ray structures of these variants (Figure 2C) where the position of the nucleophilic nitrogen of **4** superimposes with the one from the initial structure. The S112I variant displayed the highest activity among the three variants (Table 1, entry 9). The S112F variant, although structurally very similar to S112I (Figure 2C), reached roughly half of the yield as the S112I variant (Table 1, entry 10). Reasons for this might be π‐π interactions of F112 and the catalyst. S112M (PDB ID: 6T2Z) led to two different conformations of **4** (Figure S1) and very low activities (Table 1, entry 11). While all these constructs are active, they however lack enantioselectivity.

**Table 1:** Activities and selectivities of catalysts and selected streptavidin constructs for the model Baylis‐Hillman reaction substrates **1** and **2**.

	Catalyst	Mol% Cat.	Reaction time [h]	Yield [%]^[a]^	*ee* [%]^[a]^
1	–	–	48	0	
2	DMAP	2	24	3	
3	**4**	2	24	1	
4	wt streptavidin[b]	1	24	6	
5	wt streptavidin	2	48	11	<5
6	**S112A** Q114A R121A[b]+**4**	1	24	8	
7	**S112I** Q114A R121A L124Y[b]+**4**	1	24	13	
8	**S112I** Q114A R121A L124Y+**4**	2	24	16	
9	**S112I** Q114A R121A L124Y+**4**	2	48	35	<5
10	**S112F** Q114A R121A L124Y+**4**	2	48	17	<5
11	**S112M** Q114A R121A L124Y+**4**	2	48	12	<5
12	K49N **S112I** Q114A R121A L124Y **H127Q**[b]	1	48	5	
13	K49N **S112I** Q114A R121A L124Y **H127Q**[b]+4	1	48	14

Reaction conditions: HEPES buffer (10 mM, pH 7.0), streptavidin (2.6 mol % of monomer), DMSO (20 vol%), 4‐nitrobenzaldehyde (1, 50 mM), cyclopentenone (2, 100 mM), 30 °C, orbital shaking 1000 rpm. [a] Determined by HPLC in duplicates, [b] 1.3 mol % of monomer.

**Figure 2:**
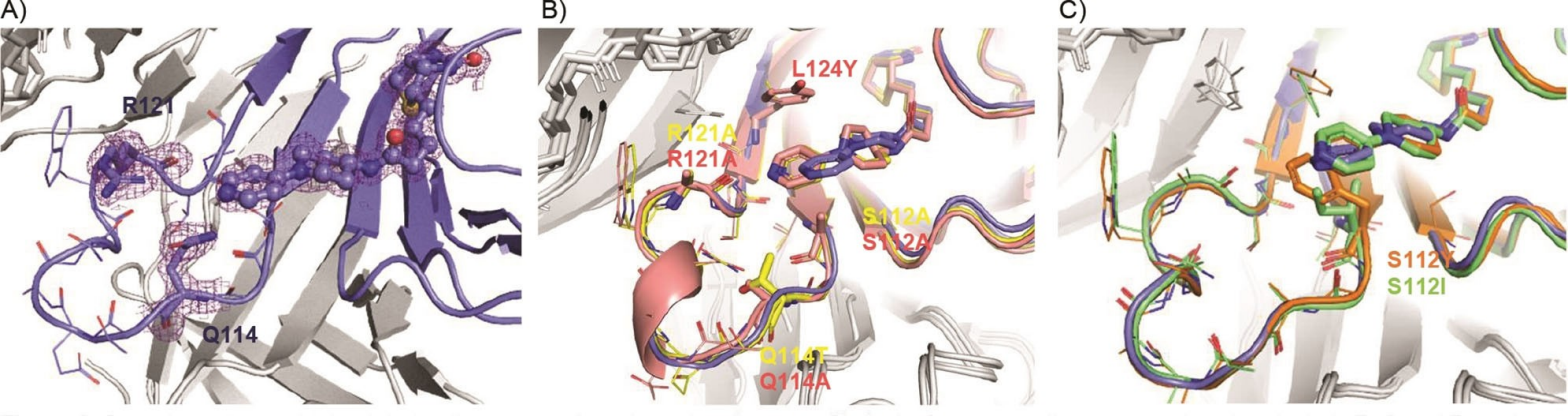
Crystal structures obtained during the course of catalyst development. Chain A of the corresponding structure is colored, chain B, C and D are in gray. A) Wt streptavidin (violet; PDB ID: 6T1E) displaying 4, blocked by residues Q114 and R121. Density obtained from the 2*mF*_o_−*DF*_c_ map. B) First and second rounds of design: S112A, Q114T and R121A (yellow, PDB ID: 6T1G) as well as S112A, Q114A, R121A, L124Y (pink, PDB ID: 6T2Y). Both variants have space at the original positions of the catalyst, but the catalyst tilted backwards into an inaccessible position. C) Third round of design in which Q114A, R121A and L124Y was accompanied by position S112 changed to F (orange, PDB ID: 6T31) or I (green, PDB ID: 6T32) leading to a good position of the catalyst within the protein environment.

Two major questions arise with these results in hand: What is the cause for the “background” reaction displayed by streptavidin alone? And why does the active protein‐cofactor system lack enantioselectivity?

We want to address the question regarding the high background first. In this context it is worth mentioning that others^[32]^ reported some proteins at high concentrations (30 mg/mL), such as the carrier protein BSA, being able to catalyze the Baylis‐Hillman reaction. However, the mechanism of catalysis was not elucidated further.

We considered histidine residues at the surface of streptavidin as additional, unwanted active sites, since it was described that (imid)azoles[^26^, ^33^] can serve as catalysts under certain conditions. Streptavidin has two histidine residues per monomer located at the surface (H87, H127; Figure S2). Both were predicted to have a p*K*_a_≤7 (SI), while the ones of all lysines are predicted above 9.8 (Table S4). As H127 is known to be tolerant against mutations,^[34]^ we created a double mutant of the S112I variant with a H127Q and K49N exchange. The latter exchange removed a lysine close to the catalyst, which could also influence the outcome of the reaction.

The yields of the background reaction dropped a bit, but remained in a similar range (Table 1, entry 12 and Table S7, entry 10). Thus, H127 has minor importance for the background reaction. The yields of the reaction with cofactor (Table 1, entry 13 and Table S7, entry 11), reveal, that K49 influences activity as the yield dropped slightly.

Exchange of H87 was already recognized to disturb the integrity of the protein.^[35]^ After careful inspection of the structures we suspect that this histidine forms a hydrogen bond to an aspartic acid (D61) of an adjacent protein chain keeping loops of neighboring subunits of streptavidin together and creating a “catalytic diad” by activating the histidine, which would explain the outcome of our experiments. Our efforts to change this H87 to a D, N or S unfortunately led to unfolded protein, only the double mutant H87Y and D61I could be refolded with very low yields and diminished biotin binding ability.

As a consequence, this variant could not be characterized further. Next, we addressed the issue of enantioselectivity. Increasing the size of the aldehyde by using Isatin^[36]^ or even bulkier *N*‐methyl‐isatin instead of nitrobenzaldehyde **1** with the aim to achieve stereoselectivity via “substrate engineering” led to yields of up to 87 %, but also in these cases no noticeable enantioselectivity was detected (Table S4). Firstly, the lack of stereocontrol was investigated more deeply using DFT methods. We employed ORCA^[37]^ to calculate a simplified reaction pathway until **I2** using a truncated cofactor 5 (SI) at a B3LYP/ma‐def2‐SVP^[39]^ level of theory using a dispersion correction^[40]^ and a CPCM model for the protein surface environment (see SI for details). To this point of the reaction pathway the chiral center is already formed^[38]^ (Figure 3A).

**Figure 3:**
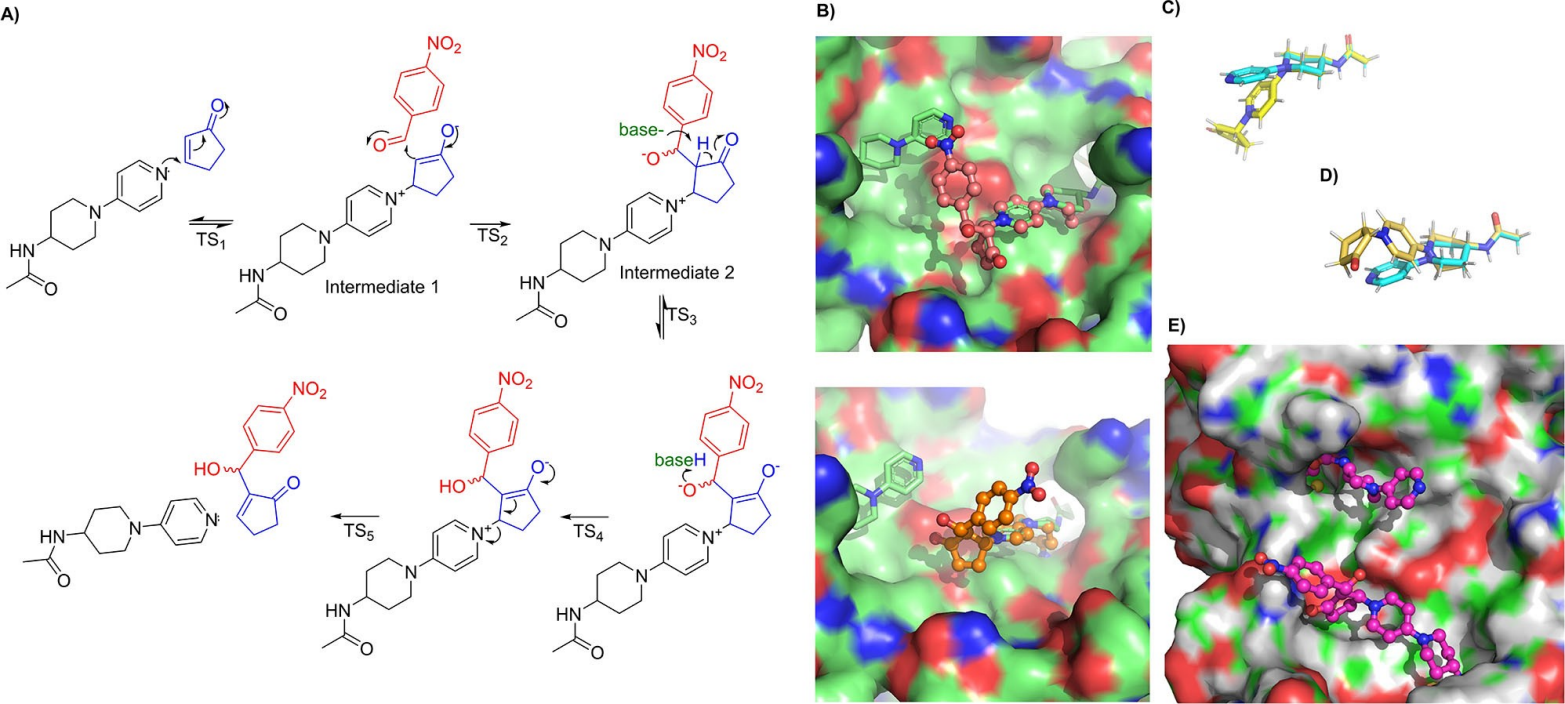
Explanation of stereoselective outcome of reaction by using DFT calculated intermediates and the protein crystal structure of streptavidin S112I Q114A R121A L124Y. A) Proposed reaction mechanism of the Baylis‐Hillman reaction. B) Both possible enantiomers of Intermediate **2** (**I2**) overlaid on the pyridine ring of catalyst **4**. Top: *R* product (pink), bottom: *S* product (orange). DFT structure of *N*‐(1‐(pyridin‐4‐yl)piperidin‐4‐yl)acetamide (turquoise) as catalyst and intermediate **1** (**I1**) in the C) chair (yellow) and D) in boat (gold) conformation overlaid on the amide bond. E) Snapshot of molecular dynamics simulation with biotin‐**I2** and streptavidin variant S112I Q114A R121A L124Y. Biotin‐**I2** has a chair conformation, and nitrobenzaldehyde **1** does not undergo any major interactions leading to a preferred stereoisomer.

By overlaying the positions of the pyridine ring of the catalyst of DFT‐derived structures and the crystal structure, one could argue that the reasons for the missing enantioselectivity are now obvious. As depicted in Figure 2B the formation of both enantiomers is possible since both intermediates are not sterically hindered by surrounding amino acids although the intermediate leading to the (*S*) product (Figure 2B, bottom) seems to undergo more favorable interactions with the protein (cyclopentenone **1** oxygen to Y124). This should at least induce a moderate enantioselectivity, which we did not observe.

There is, however, another important detail that has to be taken into account: After Michael addition of the DMAP **4** to the enone **2**, the resulting positive charge in the intermediate species (e. g., **I2** and **I3**, Figure 3A) is not primarily located at the pyridine nitrogen atom, but through mesomerism at the *para*‐nitrogen atom (Figure S3). As cofactor **4** possesses a six‐membered piperidine at this position, its conformation might change in order to accommodate a sp^2^‐hybridized iminium atom. This conformational change propagates through **4** leading to a kinked molecule. Two possible conformations of the piperidine ring are possible – either the energetically favored chair conformation^[41]^ (Figure 3C) or the unfavored (twisted) boat conformation (Figure 3D). The latter is around 3.8 kcal/mol higher in energy for **I1** using a truncated cofactor model.

Interestingly, the piperidine boat conformation of the catalyst was observed once in a solved crystal structure, namely in variant S112M Q114A R121A L124Y (PDB ID: 6T2Z, Figure S1). But only one of the two chains in the asymmetric unit harbors **4** in this conformation. The second chain has electron density for **4** in a piperidine chair conformation. Surprisingly, this variant had shown the lowest activity of all active variants.

To consider not only the small DFT model, also molecular dynamics (MD) simulations were carried out to further investigate reasons for the observed activities of these catalysts. We used the X‐ray structures of the S112F Q114A R121A L124Y and the S112I Q114A R121A L124Y variants and DFT optimized structures of biotin‐**I2** in twisted boat conformation for the *S* as well as the *R* biotin‐**I2**. Altogether, four simulations were carried out, each lasting 100 ns. While protein backbone as well as biotin displayed low RMSD values over the whole simulation, biotin‐**I2** quickly adopted a chair conformation close to the protein surface in all structures (Figures S6–S10). Therefore, we consider the chair conformation to be predominant, which might limit the efficiency of the artificial cofactor. No prevalent interactions, in particular hydrogen bonds, of **1** to the protein could be noticed in any of the simulations (Figure 3E) preferring the formation of one enantiomer over the other.

According to the presented structures and calculations the catalytically active site is too shallow to undergo interactions with **1**. As a consequence, **1** approaches **I1** in a random fashion, not interacting specifically with the protein leading to a mostly achiral product. The catalyst might undergo conformational rearrangements during the course of the reaction to accommodate the aforementioned sp^2^‐hybridized iminium atom. Hence, it should not interact too tightly with the protein, which might explain the reasons for the different activity levels of the variants harboring S112I and S122F. The catalytic rate enhancement of protein with **4** over the rate of sole **4** is most presumably due to the hydrophobic pocket around the cofactor, increasing locally the concentration of the substrates and thus facilitating the formation of **I1**.

In conclusion, a new artificial cofactor was developed, which utilizes a known organocatalyst and the biotin‐streptavidin technology to successfully catalyze the Baylis‐Hillman reaction. This protein‐cofactor system permits a yield one order of magnitude higher than the bare artificial cofactor alone. The system was evolved through repeated cycles of mutagenesis, activity tests, and structure determination. Hence, all protein design steps are rationalized. The reaction catalyzed by the artificial cofactor was further elucidated using DFT calculations and MD simulations, which identify the shallow binding pocket of the protein to be the reason for the unexpected outcome. The binding pocket especially does not allow substrate **1** to be favored in one position over the other to react with **2** in a stereoselective fashion.

Our studies clearly demonstrate that careful consideration of the course of a reaction, if fully understood, including even small changes, together with a deep knowledge of the (structural) properties of the target proteins are key to success in enzyme design. Further, we see that streptavidin, although used in many cases for the design of artificial enzymes for reasons described above, might not be a suitable scaffold for every system. In particular it lacks a deep pocket at the anchoring site of the catalyst, making it difficult to influence the stereoselective outcome of a reaction as was the case in our work.

## Conflict of interest

The authors declare no conflict of interest.

## Supporting information

As a service to our authors and readers, this journal provides supporting information supplied by the authors. Such materials are peer reviewed and may be re‐organized for online delivery, but are not copy‐edited or typeset. Technical support issues arising from supporting information (other than missing files) should be addressed to the authors.

Supplementary
